# UNDERSTANDING THE ROLE OF TASK BURDEN IN CAREGIVER NEGLECT OF PERSONS LIVING WITH DEMENTIA

**DOI:** 10.1093/geroni/igae098.2930

**Published:** 2024-12-31

**Authors:** David Hancock, E-Shien Chang, Sara Czaja

**Affiliations:** Weill Cornell Medicine, New York, New York, United States; Weill Cornell Medical College, New York, New York, United States; Weill Cornell Medicine/Center on Aging and Behavioral Research, New York, New York, United States

## Abstract

Understanding factors influencing caregivers’ perceptions of how bothersome a caregiving task is, hereafter called task burden, is vital for improving support, reducing strain, and potentially decreasing risk of caregiving neglect in persons living with dementia (PLWD). This study examines the cross-sectional associations between caregiver characteristics and perceived task burden. Data were drawn from a 6-month multicomponent, randomized controlled trial of psychosocial intervention including 244 caregivers (CG) and PLWD. Baseline data included CGs’ self-perceived task burden of 6 activities of daily living and 8 activities of instrumental activities of daily living (i.e, how much does helping PLWD with this task bother or upset you), and psycho-social assessments including depression, caregiving burden, compassion, mutualism, caregiver preparedness, spirituality, and social support. We used correlation and multivariable regression analyses to identify risk factors for task burden. Significant correlations were observed between task burden and all variables of interest. When entered simultaneously into a multivariable regression analysis, only higher depression and greater caregiving burden were independently associated with task burden (p<.05). Taken together, addressing depression and caregiving burden is crucial for reducing perceived task burden. Policy and caregiving research should prioritize interventions targeting psychological and emotional aspects of caregiving, such as mental health counseling, stress management, and caregiver support groups. Additional research should explore downstream consequences of task burden with respect to other adverse caregiving outcomes.

